# Supplementary material for: Encoding of speech in convolutional layers and the brain stem based on language experience
Source: Sci Rep. 2023 Apr 20;13:6480. doi: 10.1038/s41598-023-33384-9 (PMC10119295; doi:10.1038/s41598-023-33384-9)
Supplement: Supplementary file 1 — Supplementary Information. [file 41598_2023_33384_MOESM1_ESM.pdf]

Supplementary Materials to *Encoding of speech in convolutional layers and the brain stem based on language experience*

Gašper Beguš[1,*], Alan Zhou[1], and T. Christina Zhao[2,3]

[1]Department of Linguistics, University of California, Berkeley, United States
[2]Institute for Learning and Brain Sciences, University of Washington, United States
[3]Department of Speech and Hearing Sciences, University of Washington, United States
[*]begus@berkeley.edu

1 Supplementary Materials: Methods

1.1 Data analysis

All spectrograms are created in Praat[1] and imported into ggplot2 via a script by Matthew Winn. Peak timing was extracted with a modified script from https://stackoverflow.com/questions/48138899. .

1.2 Model Architecture

Generator		Discriminator	
Layer	Dimension	Layer	Dimension
z	100×1	input	16384×1
fc + reshape	16×1024	conv0	4096×64
upconv0	64×512	conv1	1024×128
upconv1	256×256	conv2	256×256
upconv2	1024×128	conv3	64×512
upconv3	4096×64	conv4	16×1024
upconv4	16384×1	flatten + logit	1×1

Table S1. Dimensionality of WaveGAN[2], the deep neural network examined for this experiment. Given a 100-dimensional random vector z as input, the Generator first applies a fully connected layer and reshapes the output into a 2D matrix, before applying a series of transpose convolutional layers to upsample the input into a 16834-length vector corresponding to audio. The Discriminator takes on almost a mirror architecture to the Generator, passing a 16834-length vector through a series of traditional convolutions to downsample the input, before flattening and passing the output through a fully connected layer to get the final Discriminator score.

1.3 GAMMs

A. parametric coefficients	Estimate	Std. Error	t-value	p-value
(Intercept) = English	0.1164	0.0189	6.1428	< 0.0001
LANGUAGE = Spanish	-0.0247	0.0263	-0.9388	0.3486
B. smooth terms	edf	Ref.df	F-value	p-value
s(Time)	6.1901	6.4494	9.2226	< 0.0001
s(Time):LANGUAGE = Spanish	5.7023	5.9856	2.6980	0.0147
s(Time, Subject)	243.4468	259.0000	205.5821	< 0.0001

Table S2. Estimates of a generalized additive mixed model (fitted with the *bam()* function in the *mgcv* package by[3]). Amplitude in μV from the EEG-cABR data is the dependent variable. The independent variables include LANGUAGE as a parametric predictor (with two levels, English and Spanish with English treatment-coded as the reference level), smooth for Time, by-Language difference smooth for time, and by-subject random smooths. The model includes correction for autocorrelation. Parametric coefficient LANGUAGE = Spanish estimates the overall significance in values of the cABR signal between English and Spanish (expectedly non-significant as there is no reason to expect that amplitude or base level in one of the groups would be different). The smooth term s(Time):LANGUAGE = Spanish estimates the overall significance of the difference smooth for English and Spanish and thus reveal whether the trajectories (smooths) in cABR activity between English and Spanish differ significantly. The actual difference smooth is plotted in Figure 1j.

1.4 Linear models

	Estimate	Std. Error	t value	Pr(>|t|)
(Intercept)=Period1, LanguageEnglish	-0.0010	0.0002	-4.78	0.0000
LanguageSpanish	-0.0004	0.0003	-1.59	0.1125
Place0	0.0010	0.0003	3.15	0.0017
Place2	0.0004	0.0003	1.44	0.1505
Place3	0.0006	0.0003	2.14	0.0330
Place4	0.0009	0.0003	3.24	0.0013
Place5	0.0010	0.0003	3.41	0.0007
Place6	0.0006	0.0003	1.88	0.0603
Place7	0.0013	0.0003	3.80	0.0002
Place8	0.0009	0.0005	1.74	0.0830
Place9	0.0007	0.0007	0.93	0.3512
Place10	0.0009	0.0007	1.20	0.2297
Place11	0.0011	0.0008	1.38	0.1697
Place12	0.0013	0.0008	1.64	0.1014
Replication11	-0.0008	0.0002	-3.92	0.0001
LanguageSpanish:Place0	0.0003	0.0004	0.73	0.4656
LanguageSpanish:Place2	-0.0003	0.0004	-0.67	0.5043
LanguageSpanish:Place3	0.0000	0.0004	0.05	0.9623
LanguageSpanish:Place4	-0.0005	0.0004	-1.31	0.1901
LanguageSpanish:Place5	-0.0009	0.0004	-2.22	0.0269
LanguageSpanish:Place6	0.0009	0.0004	2.10	0.0363
LanguageSpanish:Place7	-0.0004	0.0005	-0.83	0.4050
LanguageSpanish:Place8	0.0002	0.0007	0.32	0.7504
LanguageSpanish:Place9	0.0006	0.0009	0.60	0.5511
LanguageSpanish:Place10	0.0019	0.0011	1.66	0.0979
LanguageSpanish:Place11	-0.0006	0.0012	-0.47	0.6360
LanguageSpanish:Place12	-0.0005	0.0018	-0.30	0.7609
LanguageSpanish:Replication11	0.0007	0.0003	2.58	0.0101
Place0:Replication11	0.0006	0.0003	1.84	0.0657
Place2:Replication11	0.0009	0.0003	3.08	0.0022
Place3:Replication11	0.0006	0.0003	2.25	0.0248
Place4:Replication11	0.0007	0.0003	2.39	0.0170
Place5:Replication11	0.0008	0.0003	2.70	0.0071
Place6:Replication11	0.0006	0.0003	2.01	0.0449
Place7:Replication11	0.0006	0.0003	1.92	0.0548
Place8:Replication11	0.0010	0.0005	1.89	0.0593
Place9:Replication11	0.0010	0.0007	1.35	0.1773
Place10:Replication11	0.0010	0.0007	1.33	0.1837
Place11:Replication11	0.0007	0.0008	0.93	0.3547
Place12:Replication11	0.0009	0.0008	1.20	0.2315
LanguageSpanish:Place0:Replication11	-0.0003	0.0004	-0.68	0.4993
LanguageSpanish:Place2:Replication11	-0.0009	0.0004	-2.21	0.0278
LanguageSpanish:Place3:Replication11	-0.0006	0.0004	-1.52	0.1285
LanguageSpanish:Place4:Replication11	-0.0014	0.0004	-3.42	0.0007
LanguageSpanish:Place5:Replication11	-0.0017	0.0004	-4.21	0.0000
LanguageSpanish:Place6:Replication11	-0.0007	0.0004	-1.67	0.0950
LanguageSpanish:Place7:Replication11	-0.0011	0.0005	-2.20	0.0285
LanguageSpanish:Place8:Replication11	-0.0011	0.0007	-1.56	0.1186
LanguageSpanish:Place9:Replication11	-0.0008	0.0009	-0.87	0.3831
LanguageSpanish:Place10:Replication11	-0.0025	0.0011	-2.21	0.0273
LanguageSpanish:Place11:Replication11	0.0000	0.0012	0.03	0.9766

Table S3. Estimates of the linear model described in Section 4.1 (main paper) with three predictors (LANGUAGE, nTH PERIOD, and REPLICATION with all two-way and three-way interactions) for the Generator network.

	Estimate	Std. Error	t value	Pr(>|t|)
(Intercept)=Period1, LanguageEnglish	-0.0001	0.0001	-0.94	0.3497
LanguageSpanish	-0.0000	0.0002	-0.05	0.9601
Place0	0.0005	0.0002	2.29	0.0227
Place2	-0.0004	0.0002	-2.02	0.0441
Place3	-0.0001	0.0002	-0.49	0.6273
Place4	-0.0001	0.0002	-0.36	0.7177
Place5	0.0001	0.0002	0.35	0.7247
Place6	-0.0001	0.0002	-0.64	0.5257
Place7	-0.0001	0.0002	-0.58	0.5624
Place8	0.0001	0.0003	0.35	0.7287
Place9	-0.0000	0.0005	-0.10	0.9208
Place10	0.0003	0.0005	0.75	0.4510
Place11	0.0000	0.0005	0.08	0.9331
Place12	-0.0002	0.0005	-0.41	0.6834
Replication11	-0.0003	0.0001	-2.34	0.0195
LanguageSpanish:Place0	0.0000	0.0003	0.03	0.9739
LanguageSpanish:Place2	0.0003	0.0003	1.19	0.2343
LanguageSpanish:Place3	-0.0006	0.0003	-2.25	0.0248
LanguageSpanish:Place4	-0.0000	0.0003	-0.03	0.9729
LanguageSpanish:Place5	-0.0001	0.0003	-0.49	0.6212
LanguageSpanish:Place6	0.0008	0.0003	2.96	0.0032
LanguageSpanish:Place7	0.0001	0.0003	0.28	0.7768
LanguageSpanish:Place8	-0.0000	0.0004	-0.01	0.9955
LanguageSpanish:Place9	0.0001	0.0006	0.12	0.9050
LanguageSpanish:Place10	0.0011	0.0007	1.57	0.1162
LanguageSpanish:Place11	-0.0009	0.0008	-1.23	0.2205
LanguageSpanish:Place12	0.0007	0.0011	0.62	0.5364
LanguageSpanish:Replication11	-0.0000	0.0002	-0.13	0.8998
Place0:Replication11	0.0002	0.0002	1.18	0.2379
Place2:Replication11	0.0003	0.0002	1.42	0.1560
Place3:Replication11	0.0001	0.0002	0.35	0.7246
Place4:Replication11	-0.0000	0.0002	-0.02	0.9816
Place5:Replication11	-0.0000	0.0002	-0.24	0.8091
Place6:Replication11	0.0001	0.0002	0.33	0.7442
Place7:Replication11	0.0000	0.0002	0.19	0.8462
Place8:Replication11	0.0002	0.0003	0.60	0.5470
Place9:Replication11	0.0001	0.0005	0.11	0.9095
Place10:Replication11	-0.0004	0.0005	-0.81	0.4208
Place11:Replication11	-0.0001	0.0005	-0.14	0.8863
Place12:Replication11	0.0001	0.0005	0.29	0.7740
LanguageSpanish:Place0:Replication11	0.0003	0.0003	0.88	0.3769
LanguageSpanish:Place2:Replication11	0.0002	0.0003	0.81	0.4198
LanguageSpanish:Place3:Replication11	0.0004	0.0003	1.48	0.1384
LanguageSpanish:Place4:Replication11	0.0006	0.0003	2.31	0.0215
LanguageSpanish:Place5:Replication11	0.0006	0.0003	2.27	0.0236
LanguageSpanish:Place6:Replication11	0.0011	0.0003	3.76	0.0002
LanguageSpanish:Place7:Replication11	0.0005	0.0003	1.51	0.1308
LanguageSpanish:Place8:Replication11	0.0000	0.0004	0.06	0.9537
LanguageSpanish:Place9:Replication11	0.0002	0.0006	0.35	0.7262
LanguageSpanish:Place10:Replication11	-0.0005	0.0007	-0.65	0.5168
LanguageSpanish:Place11:Replication11	0.0015	0.0008	1.98	0.0483

Table S4. Estimates of the linear model described in Section 4.2 (main paper) with three predictors (LANGUAGE, nTH PERIOD, and REPLICATION with all two-way and three-way interactions) for the Discriminator network.

1.5 Individual replications

1.5.1 Generator

In the Generator network, peak latency differs significantly in periods 2, 4, 5 and 7 in the first replication and in periods 1 and 10 in the second replication (unadjusted). If adjusted with FDR, period 4 and 5 are significant in the first replication, and period 1 in the second replication (Table S5).

Place	Replication1	contrast	estimate	SE	df	t.ratio	p.value
0	1	English - Spanish	-0.0003	0.0005	517	-0.612	0.7588
1	1	English - Spanish	-0.0003	0.0004	517	-0.718	0.7237
2	1	English - Spanish	0.0009	0.0004	517	2.220	0.1164
3	1	English - Spanish	0.0003	0.0004	517	0.785	0.7029
4	1	English - Spanish	0.0016	0.0004	517	4.150	0.0005
5	1	English - Spanish	0.0023	0.0004	517	5.897	<.0001
6	1	English - Spanish	-0.0005	0.0004	517	-1.056	0.6884
7	1	English - Spanish	0.0012	0.0005	517	2.317	0.1088
8	1	English - Spanish	0.0006	0.0010	517	0.591	0.7588
9	1	English - Spanish	-0.0000	0.0014	517	-0.015	1.0000
10	1	English - Spanish	0.0004	0.0017	517	0.204	0.9611
11	1	English - Spanish	0.0002	0.0017	517	0.142	0.9611
12	1	English - Spanish	0.0003	0.0017	517	0.152	0.9611
0	2	English - Spanish	0.0005	0.0005	517	1.127	0.6763
1	2	English - Spanish	0.0012	0.0004	517	2.890	0.0348
2	2	English - Spanish	0.0006	0.0004	517	1.383	0.5548
3	2	English - Spanish	0.0006	0.0004	517	1.372	0.5548
4	2	English - Spanish	0.0003	0.0004	517	0.791	0.7029
5	2	English - Spanish	0.0004	0.0004	517	0.855	0.7029
6	2	English - Spanish	-0.0005	0.0005	517	-0.950	0.7029
7	2	English - Spanish	0.0005	0.0006	517	0.806	0.7029
8	2	English - Spanish	-0.0001	0.0008	517	-0.164	0.9611
9	2	English - Spanish	-0.0002	0.0011	517	-0.184	0.9611
10	2	English - Spanish	-0.0033	0.0014	517	-2.367	0.1088
11	2	English - Spanish	0.0018	0.0015	517	1.174	0.6763
12	2	English - Spanish					NA

P value adjustment: fdr method for 26 tests

Table S5. Pairwise contrasts in peak timing difference between English and Spanish across replications in the Generator network with FDR adjustment (with *emmeans* package by[4]). The burst is marked by the 0th period. The 12th period is not estimated due to lack of data.

1.5.2 Discriminator

In the Discriminator, periods 4 and 6 are significant in the first replication and periods 3, 4, 5 and 11 in the second replication (unadjusted). If adjusted with FDR, period 6 is significant in the first replication and period 3 in the second replication (Table S6).

Place	Replication1	contrast	estimate	SE	df	t.ratio	p.value
0	1	English - Spanish	-0.0002	0.0003	517	-0.726	0.8601
1	1	English - Spanish	0.0000	0.0002	517	0.127	1.0000
2	1	English - Spanish	-0.0005	0.0002	517	-1.915	0.2044
3	1	English - Spanish	0.0002	0.0002	517	0.908	0.7891
4	1	English - Spanish	-0.0005	0.0002	517	-2.211	0.1224
5	1	English - Spanish	-0.0004	0.0003	517	-1.697	0.2606
6	1	English - Spanish	-0.0018	0.0003	517	-6.586	<.0001
7	1	English - Spanish	-0.0005	0.0003	517	-1.595	0.2892
8	1	English - Spanish	0.0000	0.0006	517	0.013	1.0000
9	1	English - Spanish	-0.0002	0.0009	517	-0.273	1.0000
10	1	English - Spanish	-0.0006	0.0011	517	-0.580	0.8601
11	1	English - Spanish	-0.0005	0.0011	517	-0.484	0.9081
12	1	English - Spanish	-0.0007	0.0011	517	-0.606	0.8601
0	2	English - Spanish	0.0002	0.0003	517	0.741	0.8601
1	2	English - Spanish	-0.0000	0.0003	517	-0.053	1.0000
2	2	English - Spanish	-0.0001	0.0003	517	-0.428	0.9151
3	2	English - Spanish	0.0009	0.0003	517	3.635	0.0040
4	2	English - Spanish	0.0006	0.0003	517	2.200	0.1224
5	2	English - Spanish	0.0007	0.0003	517	2.561	0.0813
6	2	English - Spanish	0.0002	0.0003	517	0.646	0.8601
7	2	English - Spanish	0.0004	0.0004	517	0.946	0.7891
8	2	English - Spanish	0.0000	0.0005	517	0.029	1.0000
9	2	English - Spanish	0.0001	0.0007	517	0.182	1.0000
10	2	English - Spanish	-0.0016	0.0009	517	-1.864	0.2044
11	2	English - Spanish	0.0024	0.0010	517	2.506	0.0813
12	2	English - Spanish					NA

P value adjustment: fdr method for 26 tests

Table S6. Pairwise contrasts in peak timing difference between English and Spanish across replications in the Discriminator network with FDR adjustment (with *emmeans* package by[4]). The burst is marked by the 0th period. The 12th period is not estimated due to lack of data.

2 Without absolute values

Contrast	*n*th period	Estimate	SE	df	t.ratio	p.value
English - Spanish	0 (=burst)	0.0000	0.0004	514	0.02	0.981
English - Spanish	1	-0.0006	0.0003	514	-1.91	0.056
English - Spanish	2	-0.0007	0.0003	514	-2.06	0.040
English - Spanish	3	-0.0008	0.0003	514	-2.54	0.011
English - Spanish	4	-0.0010	0.0003	514	-2.83	0.005
English - Spanish	5	-0.0006	0.0004	514	-1.63	0.104
English - Spanish	6	-0.0017	0.0004	514	-4.13	0.000
English - Spanish	7	-0.0005	0.0005	514	-1.09	0.278
English - Spanish	8	-0.0005	0.0008	514	-0.70	0.485
English - Spanish	9	-0.0011	0.0011	514	-1.00	0.318
English - Spanish	10	-0.0021	0.0013	514	-1.63	0.104
English - Spanish	11	-0.0012	0.0014	514	-0.89	0.375

Table S7. Pairwise contrasts in peak timing difference between English and Spanish (pooled across replications) in the Generator network (with *emmeans* package[4]) if the waveforms are not converted absolute values. The burst is marked by the 0th period. The 12th period is not estimated due to lack of data.

Contrast	nth period	Estimate	SE	df	t.ratio	p.value
English - Spanish	0	-0.0001	0.0003	514	-0.33	0.745
English - Spanish	1	-0.0007	0.0003	514	-2.64	0.008
English - Spanish	2	-0.0016	0.0003	514	-5.64	0.000
English - Spanish	3	-0.0004	0.0003	514	-1.33	0.183
English - Spanish	4	-0.0019	0.0003	514	-6.77	0.000
English - Spanish	5	-0.0018	0.0003	514	-6.10	0.000
English - Spanish	6	-0.0020	0.0003	514	-5.93	0.000
English - Spanish	7	-0.0012	0.0004	514	-2.87	0.004
English - Spanish	8	-0.0007	0.0006	514	-1.18	0.239
English - Spanish	9	-0.0010	0.0009	514	-1.14	0.253
English - Spanish	10	-0.0018	0.0011	514	-1.66	0.097
English - Spanish	11	-0.0013	0.0011	514	-1.13	0.259

Table S8. Pairwise contrasts in peak timing difference between English and Spanish (pooled across replications) in the Discriminator network (with *emmeans* package[4]) if the waveforms are not converted to absolute values. The burst is marked by the 0th period. The 12th period is not estimated due to lack of data.

	Estimate	Std. Error	t value	Pr(>|t|)
(Intercept)=Period1, LanguageEnglish	-0.0010	0.0002	-4.03	0.0001
LanguageSpanish	0.0006	0.0003	1.91	0.0561
Period0	0.0009	0.0004	2.47	0.0137
Period2	0.0003	0.0003	0.89	0.3729
Period3	0.0006	0.0003	1.77	0.0778
Period4	0.0009	0.0003	2.73	0.0065
Period5	0.0010	0.0003	2.76	0.0060
Period6	0.0004	0.0004	1.10	0.2731
Period7	0.0012	0.0004	2.99	0.0030
Period8	0.0007	0.0006	1.17	0.2444
Period9	0.0007	0.0008	0.79	0.4314
Period10	0.0005	0.0008	0.56	0.5787
Period11	0.0011	0.0009	1.16	0.2464
Period12	0.0013	0.0009	1.38	0.1667
Replication11	-0.0008	0.0002	-3.31	0.0010
LanguageSpanish:Period0	-0.0006	0.0005	-1.22	0.2212
LanguageSpanish:Period2	0.0000	0.0005	0.10	0.9188
LanguageSpanish:Period3	0.0002	0.0005	0.43	0.6647
LanguageSpanish:Period4	0.0003	0.0005	0.67	0.5047
LanguageSpanish:Period5	-0.0001	0.0005	-0.13	0.8942
LanguageSpanish:Period6	0.0010	0.0005	1.95	0.0514
LanguageSpanish:Period7	-0.0001	0.0006	-0.18	0.8574
LanguageSpanish:Period8	-0.0001	0.0008	-0.14	0.8897
LanguageSpanish:Period9	0.0004	0.0011	0.38	0.7075
LanguageSpanish:Period10	0.0015	0.0014	1.11	0.2682
LanguageSpanish:Period11	0.0006	0.0014	0.41	0.6831
LanguageSpanish:Period12	0.0002	0.0021	0.11	0.9123
LanguageSpanish:Replication11	0.0009	0.0003	2.82	0.0050
Period0:Replication11	0.0005	0.0004	1.37	0.1704
Period2:Replication11	0.0008	0.0003	2.27	0.0233
Period3:Replication11	0.0006	0.0003	1.86	0.0631
Period4:Replication11	0.0007	0.0003	2.02	0.0439
Period5:Replication11	0.0008	0.0003	2.30	0.0218
Period6:Replication11	0.0007	0.0004	1.98	0.0478
Period7:Replication11	0.0006	0.0004	1.37	0.1725
Period8:Replication11	0.0009	0.0006	1.43	0.1531
Period9:Replication11	0.0010	0.0008	1.14	0.2548
Period10:Replication11	0.0013	0.0008	1.58	0.1142
Period11:Replication11	0.0007	0.0009	0.78	0.4347
Period12:Replication11	0.0009	0.0009	1.01	0.3125
LanguageSpanish:Period0:Replication11	-0.0005	0.0005	-0.87	0.3839
LanguageSpanish:Period2:Replication11	-0.0007	0.0005	-1.54	0.1231
LanguageSpanish:Period3:Replication11	-0.0007	0.0005	-1.57	0.1174
LanguageSpanish:Period4:Replication11	-0.0008	0.0005	-1.72	0.0866
LanguageSpanish:Period5:Replication11	-0.0013	0.0005	-2.59	0.0099
LanguageSpanish:Period6:Replication11	-0.0010	0.0005	-1.91	0.0565
LanguageSpanish:Period7:Replication11	-0.0003	0.0006	-0.56	0.5788
LanguageSpanish:Period8:Replication11	-0.0006	0.0008	-0.77	0.4414
LanguageSpanish:Period9:Replication11	-0.0016	0.0011	-1.48	0.1395
LanguageSpanish:Period10:Replication11	-0.0028	0.0014	-2.09	0.0371
LanguageSpanish:Period11:Replication11	-0.0005	0.0014	-0.34	0.7316

Table S9. Estimates of the linear model described in Section 4.1 (main paper) with three predictors (LANGUAGE, nTH PERIOD, and REPLICATION with all two-way and three-way interactions) for the Generator network if the waveforms are not converted to absolute values.

	Estimate	Std. Error	t value	Pr(>|t|)
(Intercept)=Period1, LanguageEnglish	-0.0001	0.0002	-0.60	0.5485
LanguageSpanish	0.0007	0.0003	2.64	0.0084
Period0	0.0004	0.0003	1.25	0.2133
Period2	-0.0005	0.0003	-1.63	0.1027
Period3	-0.0001	0.0003	-0.36	0.7221
Period4	-0.0001	0.0003	-0.23	0.8166
Period5	0.0000	0.0003	0.12	0.9066
Period6	-0.0003	0.0003	-1.00	0.3201
Period7	-0.0002	0.0003	-0.53	0.5963
Period8	-0.0001	0.0005	-0.14	0.8912
Period9	-0.0000	0.0007	-0.06	0.9492
Period10	-0.0000	0.0007	-0.07	0.9476
Period11	0.0000	0.0008	0.05	0.9570
Period12	-0.0002	0.0008	-0.26	0.7937
Replication11	-0.0003	0.0002	-1.50	0.1334
LanguageSpanish:Period0	-0.0006	0.0004	-1.41	0.1583
LanguageSpanish:Period2	0.0008	0.0004	2.12	0.0348
LanguageSpanish:Period3	-0.0004	0.0004	-0.93	0.3503
LanguageSpanish:Period4	0.0012	0.0004	2.96	0.0032
LanguageSpanish:Period5	0.0011	0.0004	2.61	0.0093
LanguageSpanish:Period6	0.0013	0.0004	2.88	0.0042
LanguageSpanish:Period7	0.0004	0.0005	0.89	0.3757
LanguageSpanish:Period8	0.0000	0.0007	0.00	0.9963
LanguageSpanish:Period9	0.0003	0.0009	0.29	0.7703
LanguageSpanish:Period10	0.0011	0.0011	0.96	0.3368
LanguageSpanish:Period11	0.0006	0.0012	0.47	0.6382
LanguageSpanish:Period12	0.0016	0.0018	0.93	0.3513
LanguageSpanish:Replication11	0.0004	0.0003	1.29	0.1973
Period0:Replication11	0.0002	0.0003	0.54	0.5907
Period2:Replication11	0.0002	0.0003	0.57	0.5688
Period3:Replication11	0.0001	0.0003	0.18	0.8558
Period4:Replication11	-0.0000	0.0003	-0.01	0.9882
Period5:Replication11	-0.0000	0.0003	-0.05	0.9619
Period6:Replication11	0.0002	0.0003	0.55	0.5799
Period7:Replication11	-0.0001	0.0003	-0.15	0.8773
Period8:Replication11	0.0001	0.0005	0.19	0.8491
Period9:Replication11	0.0001	0.0007	0.07	0.9419
Period10:Replication11	0.0000	0.0007	0.03	0.9738
Period11:Replication11	-0.0001	0.0008	-0.09	0.9269
Period12:Replication11	0.0001	0.0008	0.18	0.8539
LanguageSpanish:Period0:Replication11	-0.0001	0.0004	-0.18	0.8596
LanguageSpanish:Period2:Replication11	0.0001	0.0004	0.16	0.8735
LanguageSpanish:Period3:Replication11	0.0004	0.0004	1.14	0.2558
LanguageSpanish:Period4:Replication11	0.0010	0.0004	2.45	0.0146
LanguageSpanish:Period5:Replication11	0.0009	0.0004	2.12	0.0343
LanguageSpanish:Period6:Replication11	0.0006	0.0004	1.41	0.1597
LanguageSpanish:Period7:Replication11	0.0008	0.0005	1.56	0.1183
LanguageSpanish:Period8:Replication11	0.0003	0.0007	0.45	0.6551
LanguageSpanish:Period9:Replication11	-0.0008	0.0009	-0.85	0.3963
LanguageSpanish:Period10:Replication11	-0.0009	0.0011	-0.83	0.4056
LanguageSpanish:Period11:Replication11	0.0008	0.0012	0.68	0.4941

Table S10. Estimates of the linear model described in Section 4.2 (main paper) with three predictors (LANGUAGE, nTH PERIOD, and REPLICATION with all two-way and three-way interactions) for the Discriminator network if the waveforms are not converted to absolute values.

2.1 Individual replications without absolute values

2.1.1 Generator

In the Generator network, peak latency differs significantly in periods 1, 3, 4, 6 in the first replication and in periods 6 and 10 in the second replication (unadjusted). If adjusted with FDR, period 1 and 6 are significant in the first replication, and period 6 in the second replication (Table S11).

Period	Replication	contrast	estimate	SE	df	t.ratio	p.value
0	1	English - Spanish	-0.0005	0.0006	514	-0.797	0.6516
1	1	English - Spanish	-0.0016	0.0005	514	-3.419	0.0177
2	1	English - Spanish	-0.0009	0.0005	514	-1.945	0.1944
3	1	English - Spanish	-0.0010	0.0005	514	-2.263	0.1043
4	1	English - Spanish	-0.0011	0.0005	514	-2.339	0.1025
5	1	English - Spanish	-0.0003	0.0005	514	-0.550	0.7569
6	1	English - Spanish	-0.0016	0.0005	514	-3.049	0.0314
7	1	English - Spanish	-0.0011	0.0006	514	-1.832	0.2197
8	1	English - Spanish	-0.0008	0.0012	514	-0.710	0.6907
9	1	English - Spanish	-0.0004	0.0017	514	-0.212	0.9407
10	1	English - Spanish	-0.0002	0.0021	514	-0.119	0.9543
11	1	English - Spanish	-0.0017	0.0021	514	-0.809	0.6516
12	1	English - Spanish	-0.0018	0.0021	514	-0.877	0.6516
0	2	English - Spanish	0.0005	0.0006	514	0.850	0.6516
1	2	English - Spanish	0.0003	0.0005	514	0.625	0.7285
2	2	English - Spanish	-0.0005	0.0005	514	-0.988	0.6474
3	2	English - Spanish	-0.0006	0.0005	514	-1.345	0.3881
4	2	English - Spanish	-0.0008	0.0005	514	-1.684	0.2413
5	2	English - Spanish	-0.0009	0.0005	514	-1.691	0.2413
6	2	English - Spanish	-0.0017	0.0006	514	-2.819	0.0434
7	2	English - Spanish	0.0001	0.0008	514	0.104	0.9543
8	2	English - Spanish	-0.0002	0.0009	514	-0.232	0.9407
9	2	English - Spanish	-0.0017	0.0013	514	-1.384	0.3881
10	2	English - Spanish	-0.0040	0.0016	514	-2.476	0.0885
11	2	English - Spanish	-0.0008	0.0018	514	-0.423	0.8328
12	2	English - Spanish					NA

P-value adjustment: FDR method for 26 tests

Table S11. Pairwise contrasts in peak timing difference between English and Spanish across replications in the Generator network with FDR adjustment (with *emmeans* package by[4]) if the waveforms are not converted to absolute values. The burst is marked by the 0th period. The 12th period is not estimated due to lack of data.

2.1.2 Discriminator

In the Discriminator, periods 1-7 are significant in the first replication and periods 2 and 6 in the second replication (unadjusted). If adjusted with FDR, periods 1-7 are significant in the first replication and period 2 in the second replication (Table S12).

Period	Replication	contrast	estimate	SE	df	t.ratio	p.value
0	1	English - Spanish	-0.0004	0.0005	514	-0.801	0.5798
1	1	English - Spanish	-0.0011	0.0004	514	-2.843	0.0151
2	1	English - Spanish	-0.0020	0.0004	514	-5.168	<.0001
3	1	English - Spanish	-0.0012	0.0004	514	-3.050	0.0104
4	1	English - Spanish	-0.0032	0.0004	514	-8.410	<.0001
5	1	English - Spanish	-0.0030	0.0004	514	-7.716	<.0001
6	1	English - Spanish	-0.0030	0.0004	514	-6.769	<.0001
7	1	English - Spanish	-0.0023	0.0005	514	-4.433	0.0001
8	1	English - Spanish	-0.0014	0.0010	514	-1.438	0.2990
9	1	English - Spanish	-0.0006	0.0014	514	-0.414	0.8411
10	1	English - Spanish	-0.0012	0.0017	514	-0.721	0.6130
11	1	English - Spanish	-0.0024	0.0017	514	-1.422	0.2990
12	1	English - Spanish	-0.0027	0.0017	514	-1.592	0.2649
0	2	English - Spanish	0.0002	0.0005	514	0.354	0.8554
1	2	English - Spanish	-0.0004	0.0004	514	-0.937	0.5044
2	2	English - Spanish	-0.0011	0.0004	514	-2.854	0.0151
3	2	English - Spanish	0.0004	0.0004	514	1.099	0.4163
4	2	English - Spanish	-0.0006	0.0004	514	-1.404	0.2990
5	2	English - Spanish	-0.0006	0.0004	514	-1.306	0.3122
6	2	English - Spanish	-0.0010	0.0005	514	-2.001	0.1327
7	2	English - Spanish	-0.0000	0.0006	514	-0.065	0.9859
8	2	English - Spanish	-0.0001	0.0008	514	-0.093	0.9859
9	2	English - Spanish	-0.0014	0.0011	514	-1.354	0.3054
10	2	English - Spanish	-0.0024	0.0014	514	-1.768	0.2017
11	2	English - Spanish	-0.0001	0.0015	514	-0.084	0.9859
12	2	English - Spanish					NA

P-value adjustment: FDR method for 26 tests

Table S12. Pairwise contrasts in peak timing difference between English and Spanish across replications in the Discriminator network with FDR adjustment (with *emmeans* package by[4]) if the waveforms are not converted to absolute values. The burst is marked by the 0th period. The 12th period is not estimated due to lack of data.

References

1. Boersma, P. & Weenink, D. Praat: doing phonetics by computer [computer program]. version 5.4.06. Retrieved 21 February 2015 from http://www.praat.org/ (2015).

2. Donahue, C., McAuley, J. J. & Puckette, M. S. Adversarial audio synthesis. In *7th International Conference on Learning Representations, ICLR 2019, New Orleans, LA, USA, May 6-9, 2019*, 1–16 (OpenReview.net, 2019).

3. Wood, S. N. Fast stable restricted maximum likelihood and marginal likelihood estimation of semiparametric generalized linear models. *J. Royal Stat. Soc. (B)* **73**, 3–36 (2011).

4. Lenth, R. *emmeans: Estimated Marginal Means, aka Least-Squares Means* (2018). R package version 1.3.0.
